# Transition from a detector-dose-based to a CNR-based cerebral angiography system: evaluation of image quality and radiation dose

**DOI:** 10.1007/s12194-026-01141-y

**Published:** 2026-09-29

**Authors:** Kota Hachiya, Hiroki Abiko, Koichi Chida, Yoshikazu Matsuda

**Affiliations:** 1 Department of Radiology, Yamagata City Hospital Saiseikan, 1-3-26, Nanukamachi, Yamagata, Yamagata 990-2481 Japan; 2 Department of Radiological Examination and Technology, Tohoku University Graduate School of Medicine, 2-1 Seiryo, Aoba, Sendai, 980-8575 Japan; 3 Division of Disaster Medicine, International Research Institute of Disaster Science, Tohoku University, 6-6-4, Aoba, Sendai, 980-8579 Japan

**Keywords:** Cerebral angiography; Radiation dose; Image quality; Contrast-to-noise ratio; Endovascular treatment

## Abstract

To evaluate the image quality and clinical radiation dose of a cerebral angiography system featuring contrast-to-noise ratio (CNR)-based exposure control for neurovascular endovascular treatment (EVT). Image quality was evaluated using a quality control phantom under fluoroscopy and digital subtraction angiography (DSA). Clinical radiation dose was retrospectively compared between a conventional angiography system using detector dose-based exposure control (61 cerebral aneurysm EVT procedures) and a new angiography system using CNR-based exposure control (99 procedures). Patient entrance reference air kerma (K_a,r_) and air kerma–area product (P_KA_) were analyzed. The new system demonstrated significantly higher observer scores than the conventional system for both fluoroscopy and DSA, including spatial resolution, low-contrast resolution, and dynamic range. The median total K_a,r_ was 1818 [1301–2744] mGy with the new system and 2283 [1580–3184] mGy with the conventional system, representing a 20% reduction (p < 0.05). Conversely, the median total P_KA_ increased by 26% (173 [120–237] vs. 137 [93–207] Gy·cm^2^, p < 0.05), but remained below the current Japanese diagnostic reference level for cerebral aneurysm EVT. Compared with the conventional system, the new angiography platform demonstrated superior image quality under phantom conditions and was associated with a 20% reduction in total K_a,r_ during cerebral aneurysm EVT. Although the total P_KA_ increased by 26%, this increase may have reflected differences in the magnification strategy between the two systems, suggesting a need for further optimization.

## Introduction

Endovascular treatment (EVT), a type of interventional radiology (IR), involves the insertion of catheters into the vasculature for diagnostic or therapeutic purposes. Although EVT is less invasive than open surgery, it involves exposure to ionizing radiation [1–6]. Owing to its minimally invasive nature, EVT has gained widespread acceptance globally, with the number of procedures increasing rapidly in recent years [7–13]. However, because of the inherent complexity of these procedures, many cases require extended procedure durations, thereby raising concerns about radiation exposure to patients [14–19]. Dose limits do not apply to patients undergoing medical radiation exposure because imposing such limits could compromise optimal medical care and potentially disadvantage patients [20, 21]. Consequently, the optimization of radiation protection is crucial during medical procedures. Notably, in IR, where the patient radiation doses tend to be higher than those in other modalities, it is imperative to conduct appropriate dose assessments and management strategies to prevent skin injuries [22].

EVT for cerebral aneurysms has rapidly gained widespread acceptance with the advent of electrically detachable coils. It is now firmly established as a minimally invasive intervention capable of yielding outcomes comparable to, or even superior to, those achieved through surgical clipping, thereby benefiting a multitude of patients [23–25]. Moreover, recent technological advancements in the field have been remarkable with the continuous development of new therapeutic devices and substantial expansion in clinical indications. Notably, the advent of flow diverter stents (FDS) and the woven endobridge (WEB) has facilitated radical treatments via EVT, even for lesions that were previously challenging to manage with conventional coil embolization [26–33]. However, ischemic complications remain a major concern in EVT for cerebral aneurysms [24]. Therefore, these innovative devices are constructed from extremely fine wires with a low metal content to reduce thrombus formation, making their visibility under fluoroscopy a critical issue [34]. Ensuring optimal image quality directly enhances procedural accuracy and safety, underscoring the importance of meticulous image quality assessment and the routine maintenance of angiography systems [35–37].

**Fig. 1:**
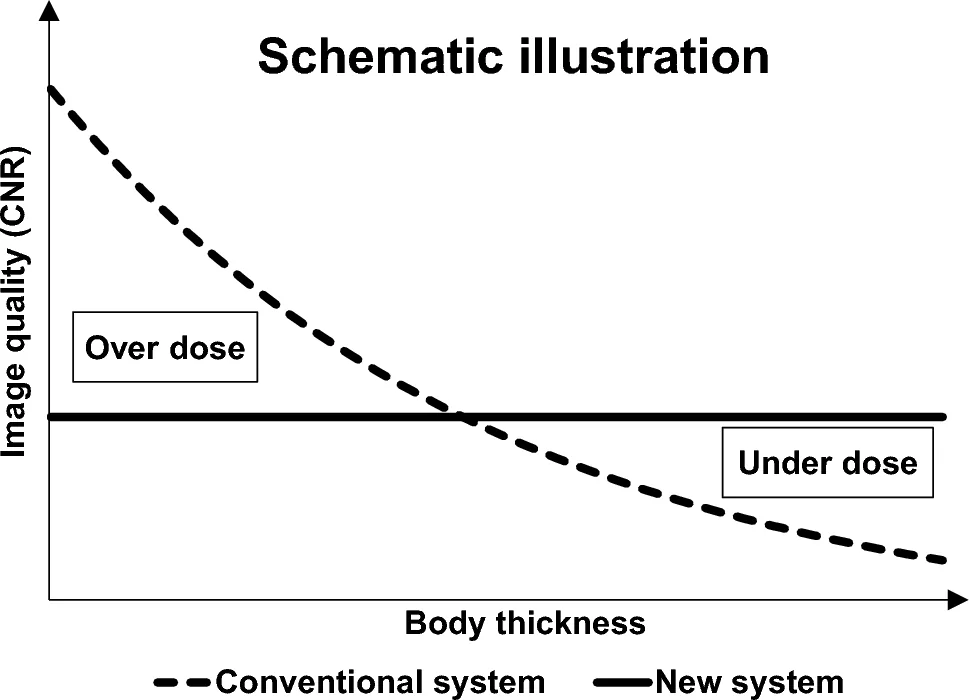
Schematic illustration comparison of exposure control concepts between the conventional detector-dose-based exposure control system and the CNR-based exposure control system. The conventional system maintains a constant detector dose, which may result in reduced image quality as body thickness increases. In contrast, the CNR-based system automatically adjusts X-ray acquisition parameters to maintain the target contrast-to-noise ratio (CNR), thereby providing more consistent image quality across different body thicknesses. This figure is intended for conceptual illustration only and does not represent experimental data

Yamagata City Hospital Stroke Center introduced the latest-generation cerebral angiography system (ARTIS icoco biplane; Siemens Healthcare GmbH, Forchheim, Germany) in January 2022. Conventional angiography systems determine the X-ray output based on the dose reaching the flat-panel detector (FPD). However, this new system incorporates a novel mechanism that observes the contrast-to-noise ratio (CNR) of the image in real-time and controls the X-ray conditions by combining all parameters to maintain a constant CNR at the lowest possible dose. Conventional exposure control systems deliver excessive X-ray exposure when the subject thickness is low or, conversely, reduce the image quality when the subject thickness increases [38]. The new system is designed to control X-ray exposure parameters to maintain consistent image quality regardless of subject thickness. (illustrative diagram: Fig. 1). It supports both fluoroscopy and digital subtraction angiography (DSA), ensuring stable image quality at an appropriate dose regardless of the patient’s build or C-arm angulation. Through phantom experiments, Werncke et al. reported that an exposure control system based on the CNR enables dose reduction while maintaining consistent image quality in the abdominal region [38]. However, in the neurovascular field, it is necessary to visualize fine intracranial vessels and treatment devices with low visibility under fluoroscopy; therefore, higher spatial resolution and lower contrast sensitivity are required compared with those needed for the abdominal region [34, 39–41]. Furthermore, because cerebral angiography involves concentrated radiation exposure to the head, optimizing the patient's radiation dose is particularly important to prevent radiation-induced skin injury.

In the neurovascular field, there have been no comprehensive clinical studies evaluating the image quality and radiation dose performance of angiographic systems equipped with a CNR-based exposure control system. Therefore, this study aimed to comprehensively evaluate the usefulness of a new cerebral angiography system equipped with a CNR-based exposure control system by conducting image quality assessments using phantom experiments and radiation dose assessments in clinical EVT cases.

## Material and methods

### Basic performance of the cerebral angiography system

**Fig. 2:**
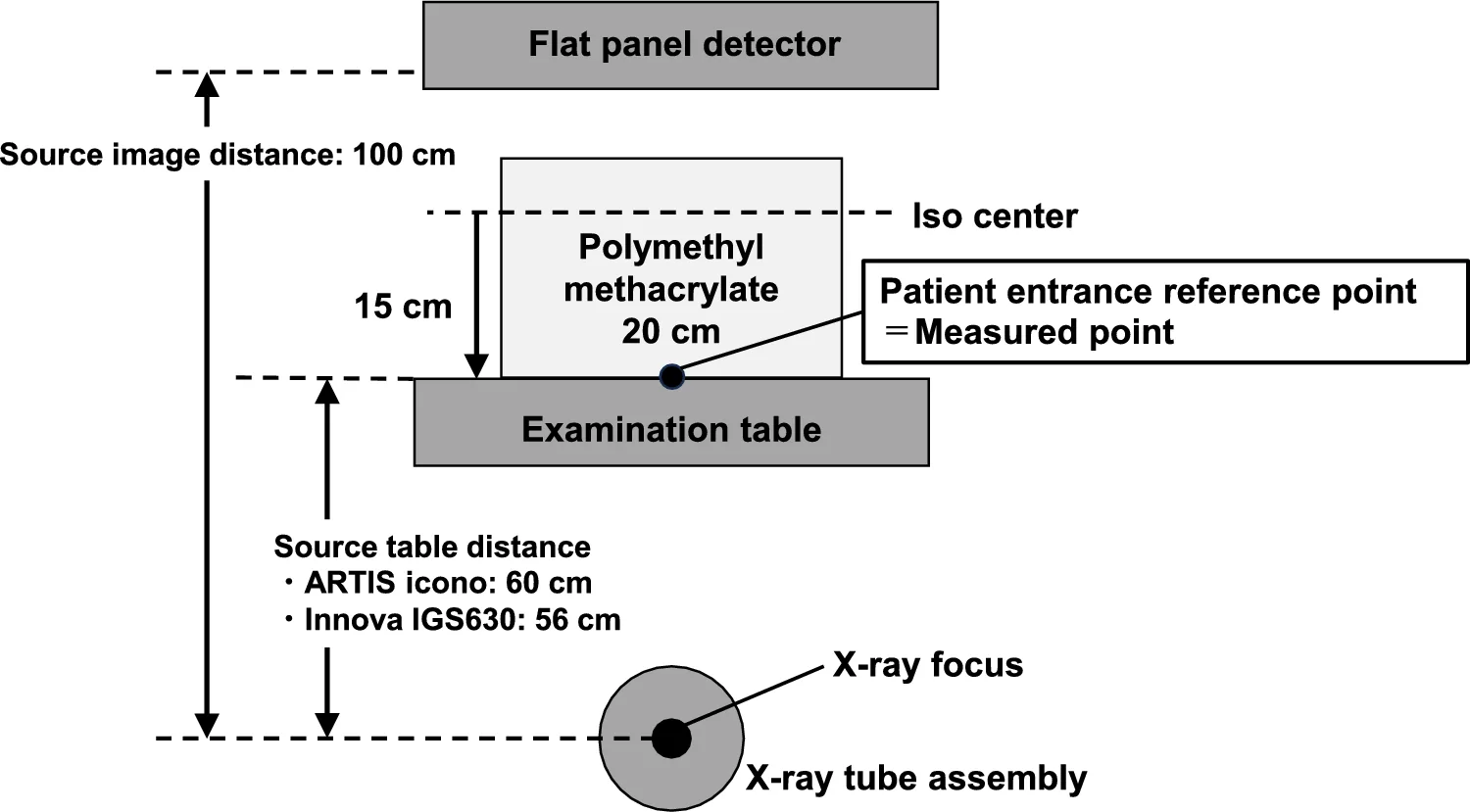
Geometric configuration for reference radiation dose measurements using a 20-cm-thick polymethyl methacrylate (PMMA) phantom. The patient entrance reference point was used as the measurement point, and the source-to-image distance was fixed at 100 cm

This study compared phantom image quality, reference radiation dose rate, and clinical radiation dose metrics between a new angiographic system featuring CNR-based exposure control and a conventional detector-dose-based system (Innova IGS630; GE HealthCare, Chicago, IL, USA). The conventional system was in clinical operation at our institution from May 2011 to December 2021. Table 1 summarizes the basic performances of the new and conventional systems and parameters most commonly used during clinical procedures. In addition, the dose at the patient entrance reference point (reference dose) was measured for both fluoroscopy and digital subtraction angiography (DSA) using both new and conventional systems. Table 2 summarizes the X-ray exposure parameters used for the measurements. The geometric configuration used during the reference dose measurements is shown in Fig. 2. The measurement methodology complied with national guidelines and involved positioning a 20-cm-thick polymethyl methacrylate (PMMA) phantom on the treatment table [42]. An Accu-Gold+ electrometer equipped with a 10X6-6 ionization chamber (Radcal Co., Monrovia, CA, USA), calibrated within the past 1 year, was used for dosimetry. The source-image distance (SID) was set to 100 cm. Both new and conventional systems operated at a fluoroscopy pulse rate of 7.5 pulses per second (pps). The DSA frame rate used clinically was variable: 4 frames per second (fps) for the first 5 s, 2 fps for the subsequent 5 s, and 1 fps thereafter. For the reference dose measurements, a frame rate of 4 fps was selected because it represents the highest frame rate in the clinical DSA protocol used in this study and corresponds to the initial acquisition setting routinely used during neurovascular EVT, making it the most clinically relevant condition. The X-ray parameters were set according to the dose modes used in routine clinical practice. For the new system, the reference doses for fluoroscopy and DSA were measured in normal and low-dose modes. These imaging protocols were configured at the time of system installation based on the manufacturer's recommended settings, which complied with the 2020 Japanese Diagnostic Reference Level (DRL) for fluoroscopy (17 mGy/min). Among the parameters affecting the X-ray exposure conditions was the image quality (IQ) level. The IQ levels in the normal-dose mode were 2.0 for fluoroscopy and 71 for DSA, whereas those in the low-dose mode were 1.4 and 56, respectively. Compared with the normal-dose mode, the IQ levels in the low-dose mode were approximately 30% lower for fluoroscopy and 27% lower for DSA. For the conventional system, the reference dose was measured only in the normal-dose mode because the low-dose mode was not used in routine clinical practice. In addition, the radiation dose during roadmap fluoroscopy was measured in the same manner. The estimated air kerma–area product (P_KA_) was then calculated by multiplying the measured dose rate by the X-ray field area. As described above, the new system automatically adjusted the X-ray parameters, including tube voltage, tube current, pulse width, additional filtration, and focal spot size, based on the CNR, whereas the conventional system adjusted these parameters based on the detector dose.

**Table 1:** Technical specifications of the new and conventional angiography systems

	ARTIS icono	Innova IGS630
Flat Panel Detector		
Matrix size (pixels)	2586 x 1904	1536 x 1536
Pixel size (μm)	154	200
Spatial resolution (lp/mm)	3.25	2.5
Gray scale resolution(bit)	16	14
Diagonal field of view	19.3/15.8/12.6*/9.8/7.9/6.3/5.2/3.9	16.7/11.1*/8.9/6.7
X-ray tube		
Anode heat storage (MHU)	5.2	3.7
Focal spot size (mm)	0.3*/0.4/0.9	0.3*/0.6/1.0
Grid ratio	15:1	13:1
Grid density (lines/cm)	80	70
Inherent filtration (mmAl)	2.5	3.5
Additional filtration (mmCu)	0.1/0.2/0.3/0.6*/0.9	0.1/0.2/0.3*
Maximum tube voltage (kV)	125	125
Maximum tube current (mA)	1000	1000

* Parameters most commonly used for cerebral aneurysm EVT in our institution

**Table 2:** X-ray exposure parameters used for reference dose measurements with a 20-cm PMMA phantom

	Fluoroscopy			Digital subtraction angiography		
	ARTIS icono		Innova IGS630	ARTIS icono		Innova IGS630
	Normal-dose	Low-dose	Normal-dose	Normal-dose	Low-dose	Normal-dose
Tube voltage (kV)	71–84	68–84	79–81	70–81	67–80	86–90
Pulse width (ms)	6.2–7.4	5.7–7.3	7.0–8.0	85–100	91–100	52–55
Additional filter (mmCu)	0.3–0.6	0.3–0.6	0.3	0.3–0.6	0.3–0.6	0.1
Pulse/Frame rate	7.5 pps	7.5 pps	7.5 pps	4 fps	4 fps	4 fps

*PMMA* polymethyl methacrylate; *pps* pulses per second; *fps* frames per second

### Image quality evaluation

**Fig. 3:**
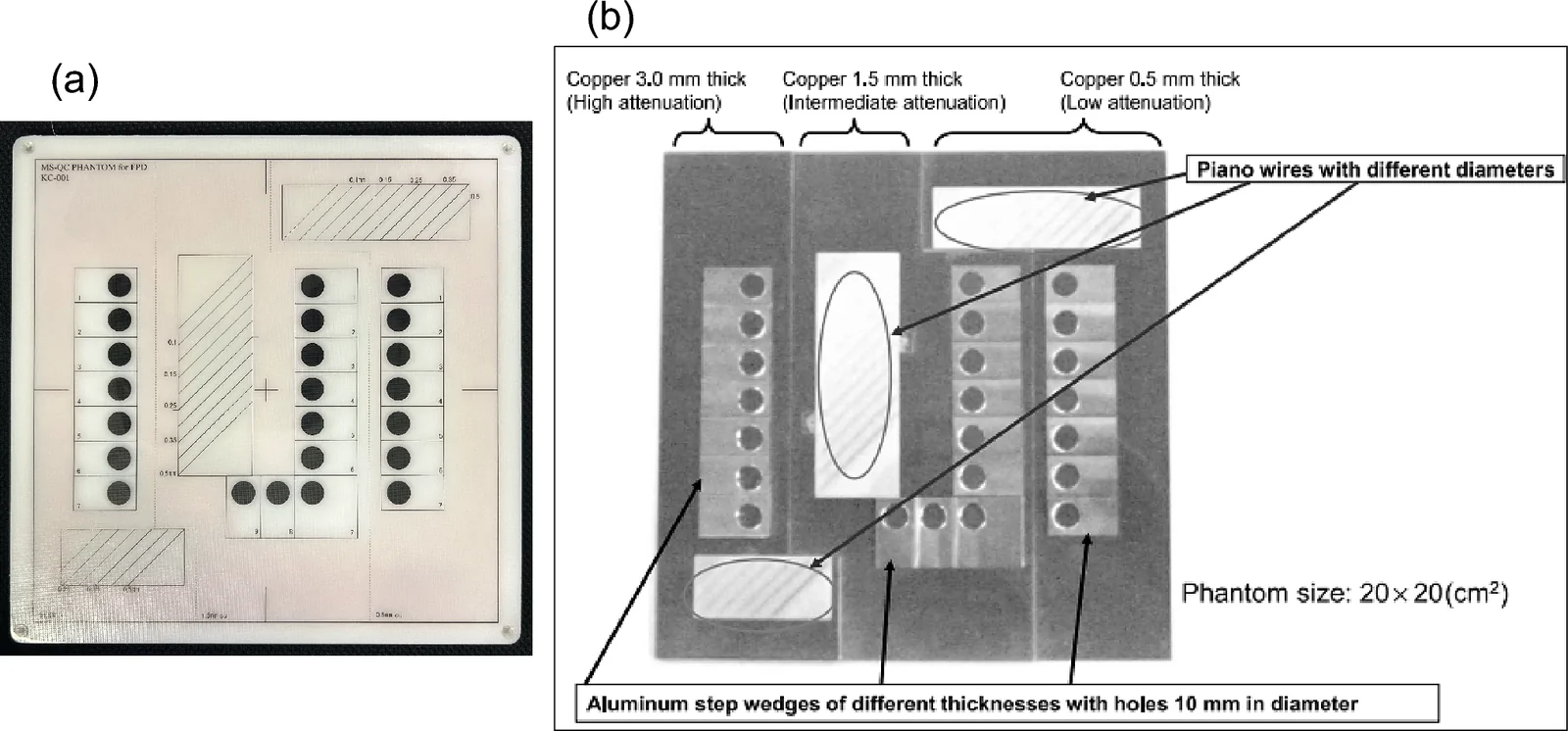
Quality control (QC) phantom (KC-001) used for image quality evaluation. **a** Appearance of the KC-001 phantom. **b** Structure of the KC-001 phantom. The phantom consists of three copper plates with thicknesses of 0.5, 1.5, and 3.0 mm, incorporating wire charts with diameters ranging from 0.08 to 0.5 mm and aluminum step wedges with thicknesses ranging from 0.1 to 2.7 mm

**Fig. 4:**
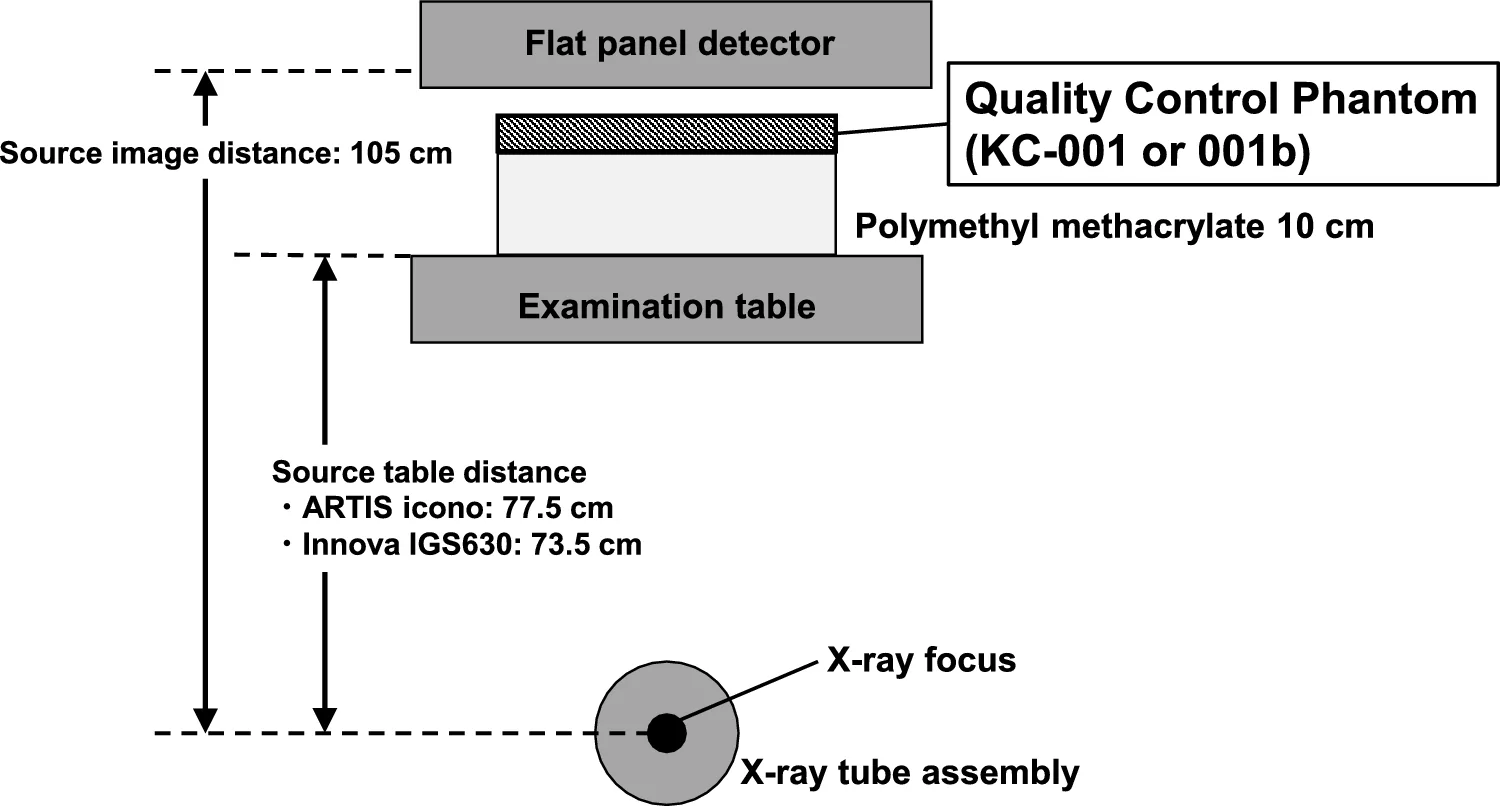
Geometric configuration for phantom image quality evaluation. The quality control (QC) phantom (KC-001 or KC-001b) was positioned on a 10-cm-thick polymethyl methacrylate (PMMA) phantom to achieve a displayed patient entrance reference air kerma (K_a,r_) comparable to that obtained with a 20-cm-thick PMMA phantom

**Fig. 5:**
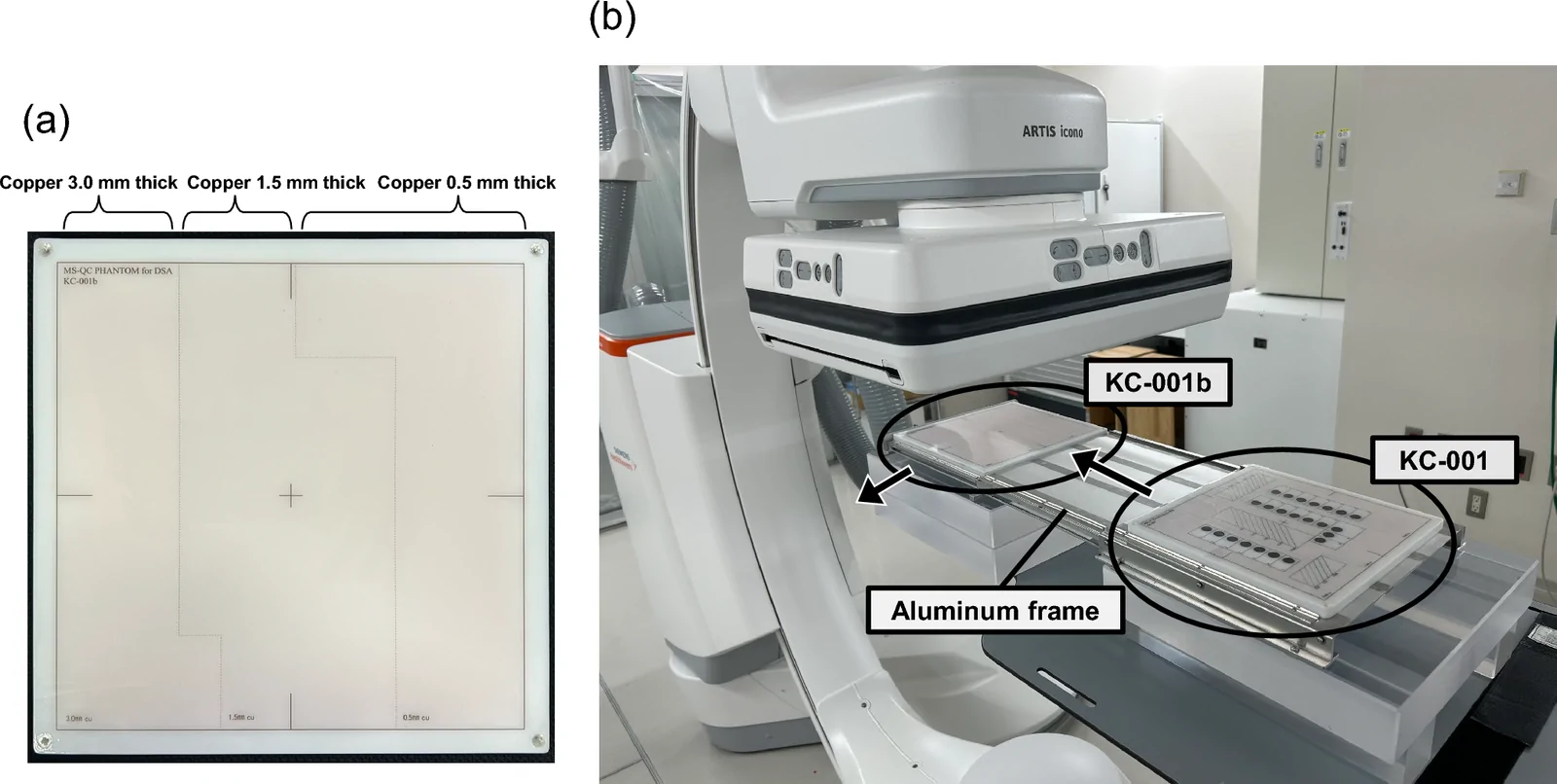
KC-001b phantom and DSA image acquisition procedure. **a** Appearance of the KC-001b phantom. The KC-001b phantom consists of copper plates with the same thicknesses as those of the KC-001 phantom but does not contain wire charts or aluminum step wedges. **b** DSA image acquisition procedure. Mask images were acquired using the KC-001b phantom and were subsequently replaced by the KC-001 phantom for contrast images. An aluminum frame was used to facilitate phantom translation and minimize misregistration

The image quality of fluoroscopy and DSA was evaluated for both new and conventional systems using FPD quality control (QC) phantoms (KC-001 and KC-001b; Mitaya Manufacturing Co., Japan). Chida et al. reported that the use of this phantom facilitated a straightforward evaluation of the spatial resolution, low-contrast resolution, and dynamic range of X-ray images [43]. The appearance and structure of the KC-001 phantom are shown in Fig. 3. The KC-001 phantom consists of three types of copper plates with varied thicknesses (0.5, 1.5, and 3.0 mm), upon which wire charts with different diameters (0.08–0.5 mm) and aluminum steps with various thicknesses (0.1–2.7 mm) are arranged. Figure 4 presents the geometric configuration used to obtain evaluation images. To obtain a radiation dose approximately equivalent to that measured using a 20-cm-thick PMMA phantom, the KC-001 phantom was placed on top of a 10-cm-thick PMMA phantom. The patient entrance reference air kerma (K_a,r_) displayed by the angiographic system was within 10% of that obtained with a 20-cm-thick PMMA phantom under all fluoroscopic and DSA conditions (Table 3). The table height was adjusted to its maximum to ensure that the entire KC phantom was included within the field of view. For DSA image acquisition, the KC-001b phantom was initially placed and mask images were captured; it was subsequently replaced with the KC-001 phantom. The KC-001b phantom is composed of copper plates of identical thickness to those of the KC-001 phantom, but it lacks a wire chart and an aluminum step wedge (Fig. 5a). Additionally, an aluminum frame was used to minimize misregistration when the KC-001 phantom was manually translated (Fig. 5b). The measured field encompassed the entire X-ray field, and the aluminum frame was included within the X-ray field. Therefore, the possibility that the aluminum frame influenced the automatic exposure control cannot be excluded. However, because the same frame configuration was used under all measurement conditions, it is unlikely that this had a substantial impact on the relative comparisons between the two systems or between the dose modes. Both fluoroscopy and DSA were performed in clinical dose mode. The new system acquired images in both normal- and low-dose modes. The conventional system acquired images only in the normal-dose mode.

**Table 3:** Comparison of displayed patient entrance reference air kerma (K_a,r_) between a 20-cm PMMA phantom and a 10-cm PMMA phantom with the KC-001 phantom

	System (Dose mode)	20-cm PMMA (mGy/min)	10-cm PMMA + KC-001 (mGy/min)	Difference (%)
Fluoroscopy	Innova IGS630 (Normal-dose)	8.7	8.8	1.2
	ARTIS icono (Normal-dose)	9.1	9.9	8.8
	ARTIS icono (Low-dose)	6.6	7.2	9.1
DSA	Innova IGS630 (Normal-dose)	290	295	1.7
	ARTIS icono (Normal-dose)	112	122	8.9
	ARTIS icono (Low-dose)	142	155	9.2

**Fig. 6:**
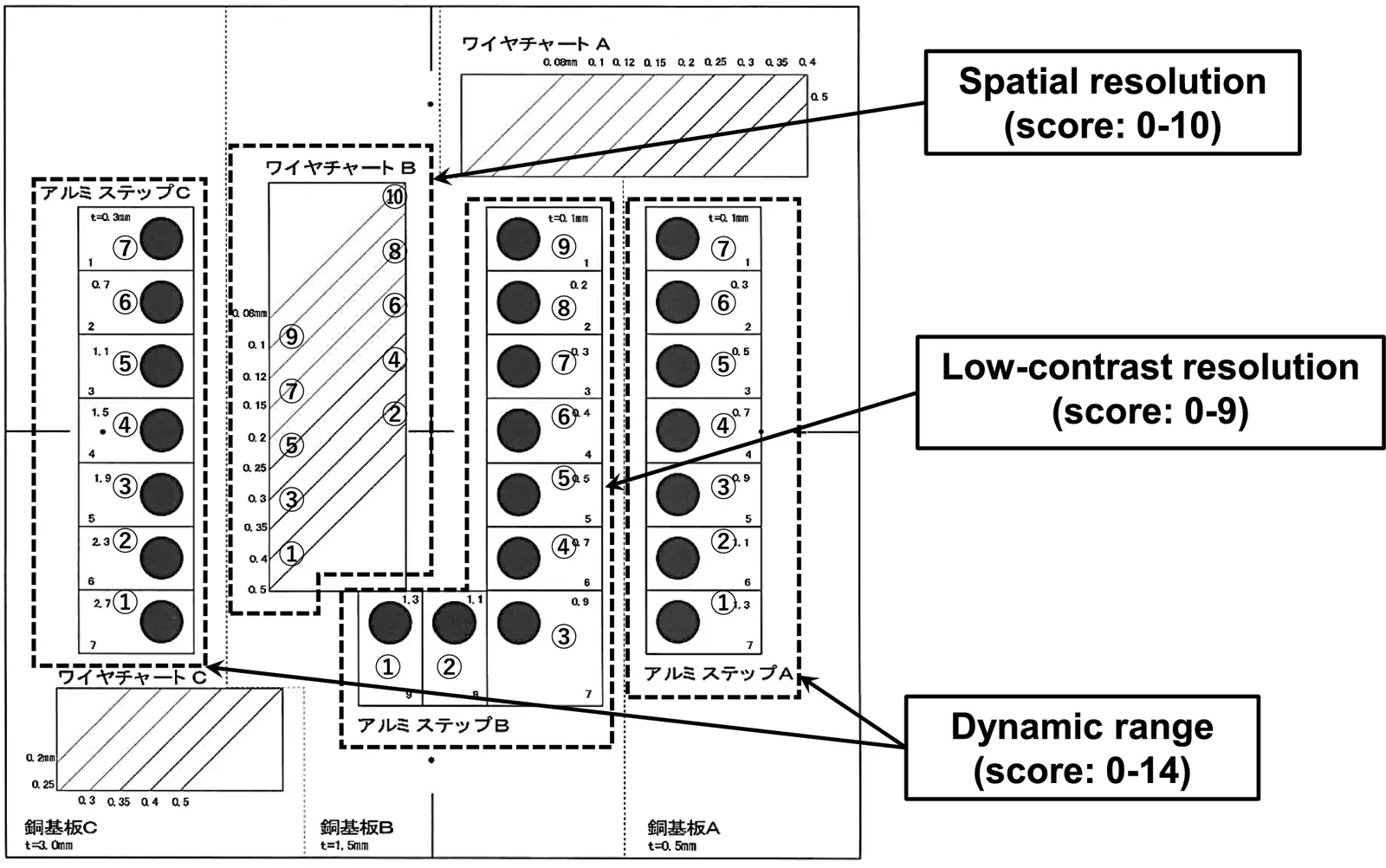
Schematic illustration of the image quality evaluation parameters. Spatial resolution, low-contrast resolution, and dynamic range were evaluated using the KC-001 phantom. Higher scores indicate better image quality

Figure 6 shows the image quality evaluation parameters and methods used in this study. Table 4 summarizes the correspondence between the KC-001 phantom scores and the physical image quality metrics. Spatial resolution was evaluated by scoring the minimum wire chart diameter distinguishable in the medium-contrast region (1.5 mm Cu) on a scale of 0–10 points (higher scores indicate improved spatial resolution). Low-contrast resolution was evaluated by scoring the minimum thickness of the aluminum steps distinguishable in the medium-contrast region on a scale of 0–9 points (higher scores indicate improved low-contrast resolution). Dynamic range was evaluated by scoring the minimum thickness of aluminum steps distinguishable in the low-contrast region (0.5 mm Cu) and the high-contrast region (3.0 mm Cu) each on a scale of 0–7 points and summing the two scores (i.e., higher scores indicate improved dynamic range). The evaluators comprised 10 individuals: 1 endovascular neurosurgery instructor, 1 specialist, and 8 radiologic technologists with over 10 years of EVT experience at our institution.

**Table 4:** Correspondence between KC-001 phantom scores and physical image quality metrics

	Score	Minimum resolvable wire diameter (mm)
Spatial resolution	10	0.06
	9	0.1
	8	0.12
	7	0.15
	6	0.2
	5	0.25
	4	0.3
	3	0.35
	2	0.4
	1	0.5

	Score	Minimum detectable aluminum step thickness (mm Al)
Low-contrast resolution	9	0.1
	8	0.2
	7	0.3
	6	0.4
	5	0.5
	4	0.7
	3	0.9
	2	1.1
	1	1.3

	Score	
Dynamic range	14	Total number of detectable aluminum steps under 0.5-mm and 3.0-mm-thick copper plates
	…	
	1	

*Al * aluminum

Image quality was evaluated using fluoroscopic and DSA video sequences displayed on the clinical monitors routinely used for neurovascular EVT in the respective angiography suites. Because the evaluations were performed under routine clinical viewing conditions, blinding of the angiography systems was not feasible. However, the order of image presentation for the three imaging conditions (new system, normal-dose mode; new system, low-dose mode; and conventional system) was randomized independently for each observer to minimize potential order effects. Image quality scores were compared between the new and conventional systems for each evaluation parameter.

### Clinical dose evaluation

Details of the cerebral aneurysm cases included in this study are shown in Table 5. This study included all EVT procedures performed at our facility for ruptured and unruptured cerebral aneurysms between January 2020 and April 2024. Treatment using FDSs or the WEB system has been reported to be associated with shorter fluoroscopy times and lower radiation exposure than adjunctive techniques [44, 45]. Because 11 procedures using the WEB system were performed exclusively with the new system, these cases were excluded from the analysis. In addition, four cases involving the treatment of two or more aneurysms were also excluded. Consequently, a total of 160 cases were analyzed, including 61 treated with the conventional system (January 2020–December 2021) and 99 treated with the new system (January 2022–April 2024). All procedures were performed under general anesthesia by two board-certified endovascular neurosurgery instructors and one specialist.

Regarding treatment techniques, the simple technique was used in 80 cases (47 with the new system and 33 with the conventional system), the adjunctive technique in 44 cases (30 and 14 cases, respectively), and FDS placement in 36 cases (22 and 14 cases, respectively).

**Table 5:** Patient characteristics of cerebral aneurysm cases included in this study

Characteristic	ARTIS icono	Innova IGS630
Period	Jan 2022- Apr 2024	Jan 2020- Dec 2021
Number of cases	99	61
Male / Female	27/ 72	11/ 50
Age, years	67 [51–77]	68 [53–79]
Ruptured / Unruptured	42/ 57	26/ 35
Aneurysm location		
ICA	48	27
MCA	12	2
ACA/ AcomA	19	10
BA	10	10
VA/ PICA	10	12
Endovascular procedures		
Simple technique	48	33
Adjunctive technique	33	14
FDS	22	14

Values are presented as number of cases unless otherwise indicated. Age is presented as median [interquartile range]

*ICA* internal carotid artery; *MCA* middle cerebral artery; *ACA* anterior cerebral artery; *AcomA* anterior communicating artery; *BA* basilar artery; *VA* vertebral artery; *PICA* posterior inferior cerebellar artery; *FDS* flow diverter stent

The K_a,r_ and P_KA_ were retrospectively obtained from the dose management software. Related parameters, including fluoroscopy time and DSA acquisition time, were also retrieved. These values represent the combined total of the frontal-plane and lateral-plane C-arms. The DSA acquisition time was defined as the duration during which the hand switch or foot pedal was activated. However, it should be noted that, because both systems used a variable DSA frame rate, this metric may not fully capture the total DSA-related radiation time. These data were compared between the two systems. Furthermore, the relationship between the total K_a,r_, which has been reported to correlate most strongly with the maximum incident skin dose in the cerebrovascular region, and related parameters was also analyzed [46, 47]. Both systems were used in the normal-dose mode for fluoroscopy and DSA in these cases. In routine clinical practice, a diagonal FOV of 11.1 inches was typically used with the conventional system, whereas a 12.6-inch FOV was typically used with the new system. During roadmap-guided device deployment, roadmap images were magnified using a 6.7-inch FOV with the conventional system, whereas the new system used digital zoom while maintaining the 12.6-inch FOV. The conventional system was not equipped with a digital zoom function. In the new system, digital zoom was available only for roadmap images and was not applied during live fluoroscopy. Consequently, collimation had to be adjusted manually during live fluoroscopy.

### Statistical analysis

Statistical analyses were performed using the EZR software (Jichi Medical University Saitama Medical Center, Saitama, Japan) [48]. The image quality scores for spatial resolution, low-contrast resolution, and dynamic range were compared among the three groups using the Kruskal–Wallis test followed by Holm's post hoc test. To assess interobserver agreement for the subjective image-quality evaluation, a rank-based intraclass correlation coefficient (ICC) was calculated using the scores assigned by the 10 observers. Clinical radiation dose metrics and related parameters were compared between the two systems using the Mann–Whitney U test. Correlation analysis was performed using the Spearman’s rank correlation coefficient. The threshold for statistical significance was set at p<0.05.

## Results

### Reference dose

**Fig. 7:**
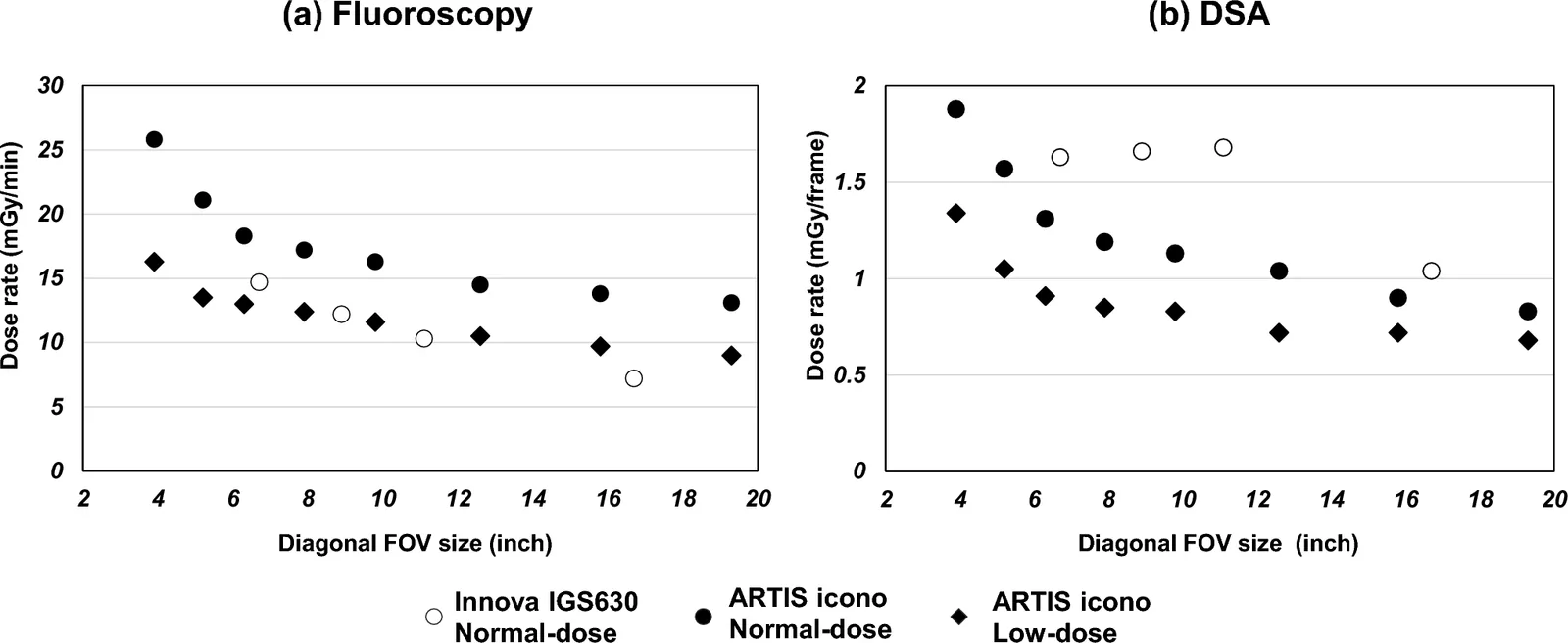
Reference radiation dose as a function of the diagonal field-of-view (FOV) size. **a** Fluoroscopic dose rate. **b** DSA dose per frame. Measurements were performed using a 20-cm-thick polymethyl methacrylate (PMMA) phantom. The new system was evaluated in both normal- and low-dose modes, whereas the conventional system was evaluated in the normal-dose mode only

**Fig. 8:**
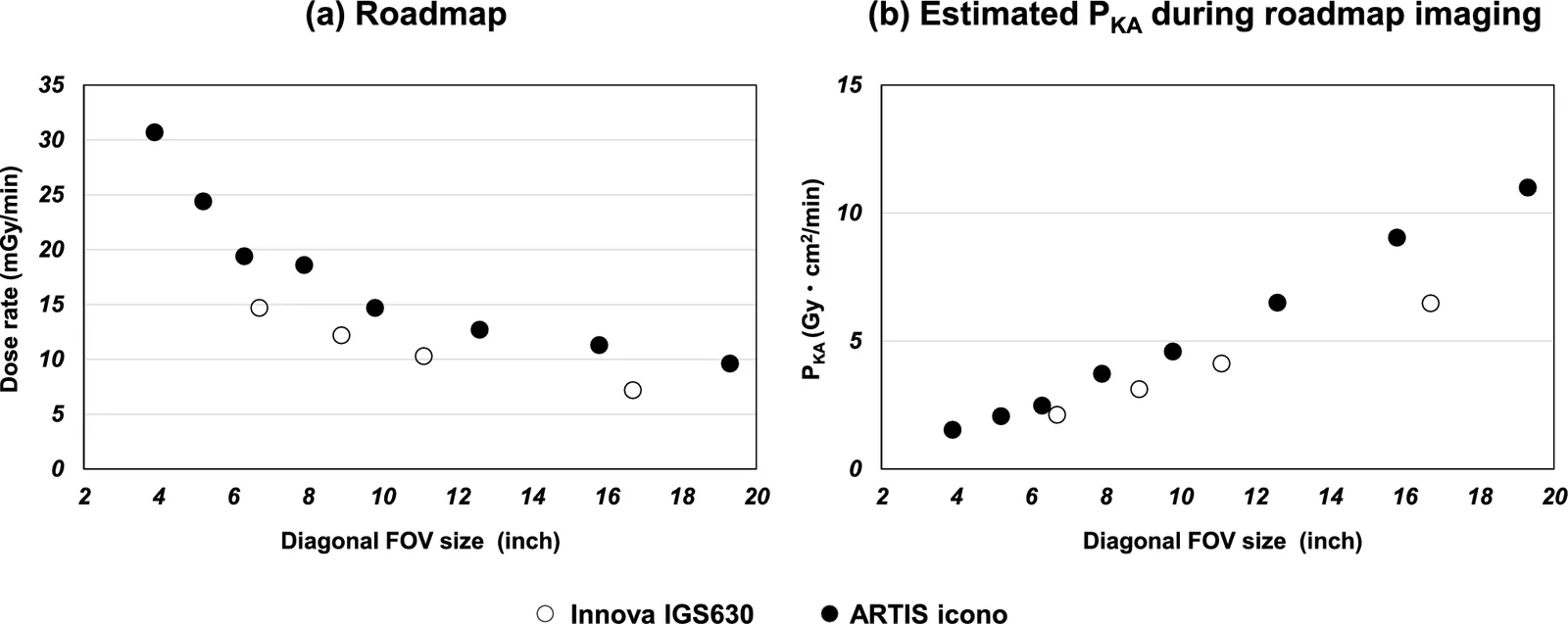
Relationship between the diagonal field-of-view (FOV) size and roadmap fluoroscopy dose. **a** Roadmap fluoroscopy dose rate. **b** Estimated air kerma–area product (PKA) during roadmap fluoroscopy, calculated from the measured dose rate and irradiated field size

Reference doses for fluoroscopy and DSA are shown in Fig. 7. For the new system, at the most frequently used FOV (12.6 inches), the reference fluoroscopic dose rate was 14.5 mGy/min in the normal-dose mode and 10.5 mGy/min in the low-dose mode. The reference DSA dose was 1.04 mGy/frame in the normal-dose mode and 0.72 mGy/frame in the low-dose mode. For the conventional system, the reference fluoroscopic dose rate was 10.3 mGy/min, and the reference DSA dose was 1.68 mGy/frame, at an FOV of 11.1 inches. The reference dose and estimated P_KA_ during roadmap fluoroscopy are shown in Fig. 8. At the field sizes most commonly used for roadmap fluoroscopy, the dose rate was 12.7 mGy/min for the new system (12.6-inch FOV) and 14.7 mGy/min for the conventional system (6.7-inch FOV). The estimated PKA during roadmap fluoroscopy was 6.5 Gy·cm^2^/min for the new system (12.6-inch FOV) and 2.1 Gy·cm^2^/min for the conventional system (6.7-inch FOV).

### Image quality evaluation

**Fig. 9:**
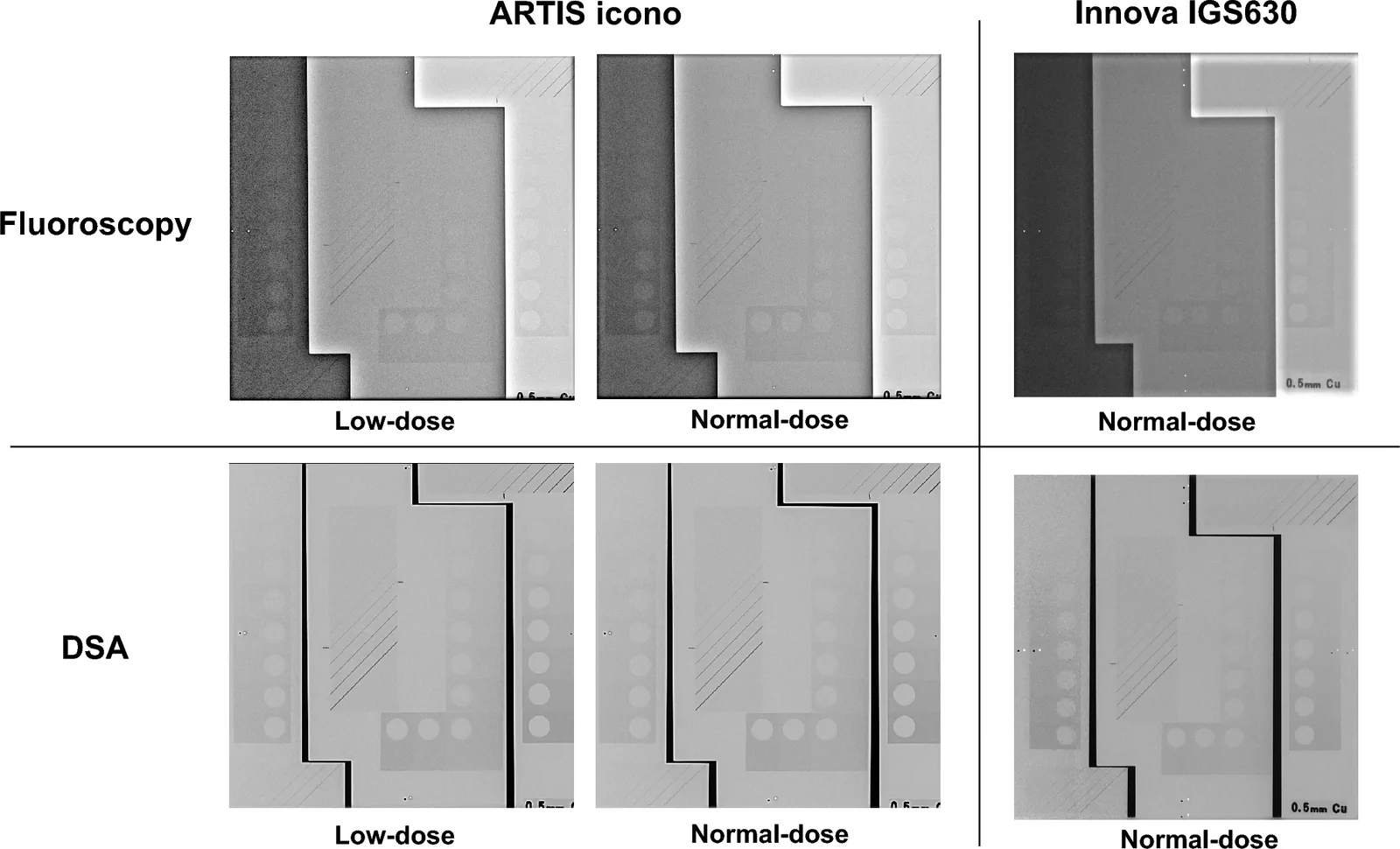
Representative phantom images used for visual image quality evaluation. Fluoroscopic (upper row) and DSA (lower row) images acquired with the new system in the low- and normal-dose modes and with the conventional system in the normal-dose mode

**Fig. 10:**
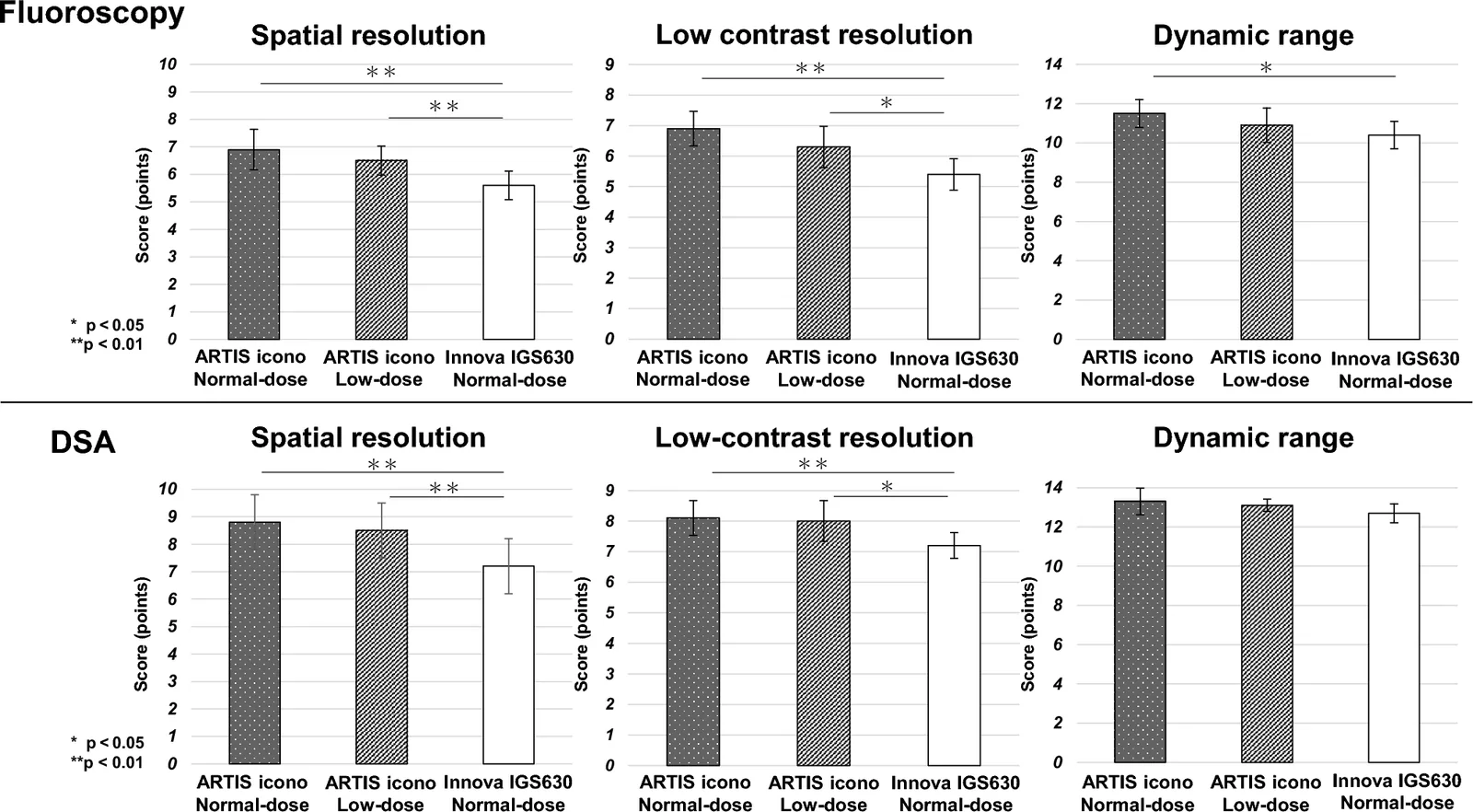
Visual image quality evaluation scores for fluoroscopic and DSA images. Mean scores for spatial resolution, low-contrast resolution, and dynamic range are shown for the new system in the normal- and low-dose modes and for the conventional system in the normal-dose mode. Error bars indicate standard deviation. Horizontal bars indicate significant differences between groups (*p < 0.05, **p < 0.01)

Representative fluoroscopy and DSA images used for the evaluation are shown in Fig. 9. The evaluation scores (expressed as mean ± standard deviation [SD]) for the fluoroscopy and DSA images in each system and dose mode, categorized by the evaluation parameters, are shown in Fig. 10. For fluoroscopic images, the spatial resolution scores were 6.9 ± 0.7 in the new system’s normal-dose mode, 6.5 ± 0.5 in the new system's low-dose mode, and 5.6 ± 0.5 in the conventional system; the corresponding low-contrast resolution scores were 6.9 ± 0.6, 6.3 ± 0.7, and 5.4 ± 0.5, respectively, and the corresponding dynamic range scores were 11.5 ± 0.7, 10.9 ± 0.9, and 10.4 ± 0.7, respectively. The image quality scores in the normal-dose mode of the new system were higher than those of the conventional system for all evaluation parameters (spatial resolution and low-contrast resolution, p < 0.01; dynamic range, p < 0.05). In addition, the low-dose mode of the new system showed higher scores than the conventional system for spatial resolution (p < 0.01) and low-contrast resolution (p < 0.05). Moreover, no statistically significant differences were observed between the normal-dose and low-dose modes of the new system for any evaluation parameters (spatial resolution: p = 0.22; low-contrast resolution: p = 0.06; dynamic range: p = 0.12).

For DSA images, the spatial resolution scores were 8.8 ± 0.4 in the new system’s normal-dose mode, 8.5 ± 0.5 in the new system’s low-dose mode, and 7.2 ± 0.4 in the conventional system; the corresponding low-contrast resolution scores were 8.1 ± 0.6, 8.0 ± 0.7, and 7.2 ± 0.4, respectively, and the corresponding dynamic range scores were 13.3 ± 0.7, 13.1 ± 0.3, and 12.7 ± 0.5, respectively. The image quality scores in the normal-dose mode of the new system were higher than those of the conventional system for spatial resolution and low-contrast resolution (p < 0.01). In addition, the low-dose mode of the new system showed higher scores than the conventional system for spatial resolution (p < 0.01) and low-contrast resolution (p < 0.05). No statistically significant differences were observed between the normal-dose and low-dose modes of the new system for any evaluation parameters (spatial resolution, p = 0.19; low-contrast resolution, p = 0.75; dynamic range, p = 0.35).

For fluoroscopic images, the rank-based ICCs were 0.84 (95% CI, 0.62–0.98), 0.76 (95% CI, 0.48–0.97), and 0.79 (95% CI, 0.52–0.97) for spatial resolution, low-contrast resolution, and dynamic range, respectively. For DSA images, the rank-based ICCs were 0.91 (95% CI, 0.68–0.99), 0.76 (95% CI, 0.37–0.99), and 0.47 (95% CI, 0.07–0.98) for spatial resolution, low-contrast resolution, and dynamic range, respectively.

### Clinical dose evaluation

**Fig. 11:**
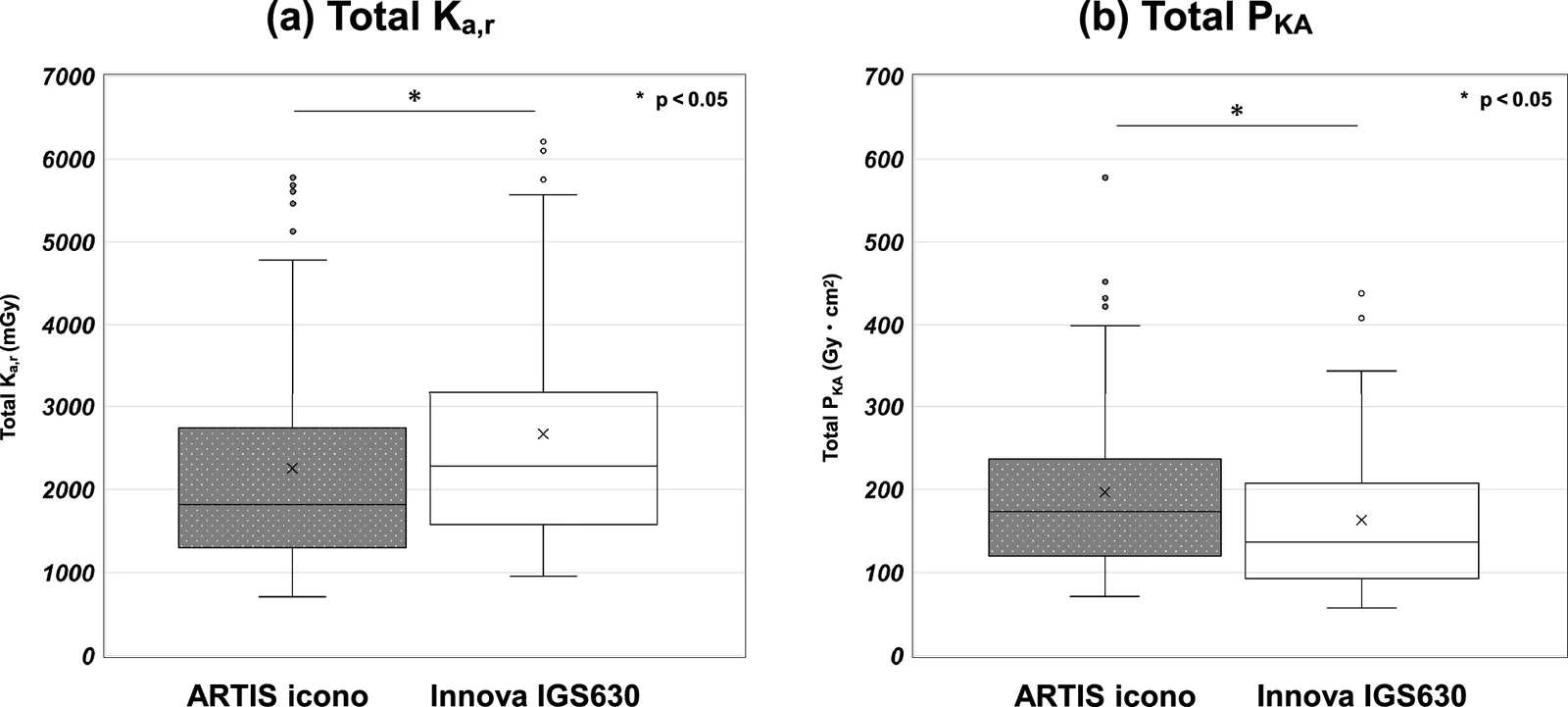
Comparison of clinical radiation dose between the new and conventional angiography systems. **a** Total patient entrance reference air kerma (Ka,r). **b** Total air kerma–area product (PKA). Boxes represent the interquartile range, the horizontal line indicates the median, whiskers indicate the minimum and maximum values excluding outliers, circles indicate outliers, and × indicates the mean. Horizontal bars indicate statistically significant differences between groups (*p < 0.05, **p < 0.01)

The total K_a,r_ and total P_KA_ (expressed as median [interquartile range]) for the cerebral aneurysm treatment cases are shown in Fig. 11. The total K_a,r_ was 1818 [1301–2744] mGy for the new system and 2283 [1580–3184] mGy for the conventional system (p < 0.05). The total P_KA_ was 173 [120–237] Gy·cm^2^ for the new system and 137 [93–207] Gy·cm^2^ for the conventional system (p < 0.05).

**Fig. 12:**
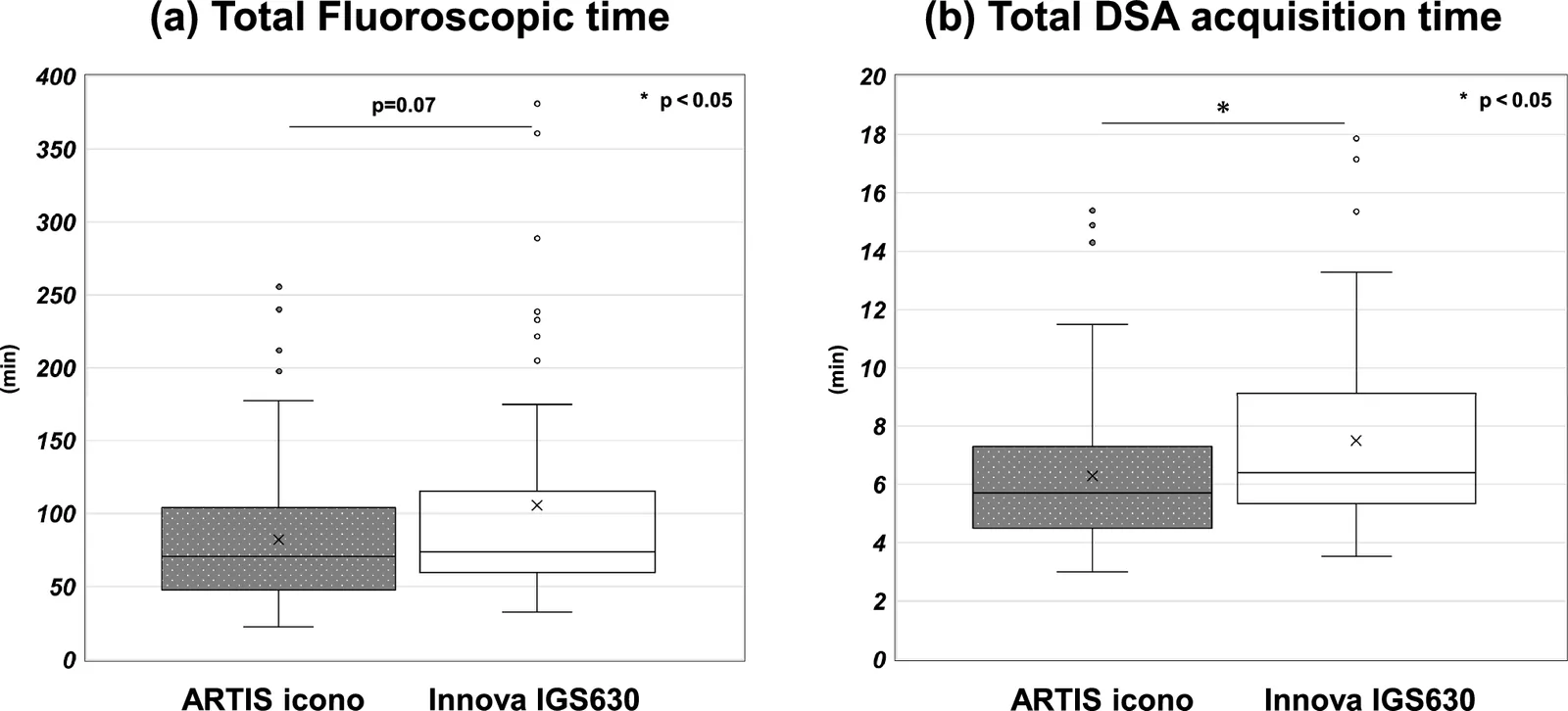
Comparison of procedural parameters between the new and conventional angiography systems. **a** Total fluoroscopy time. **b** Total DSA acquisition time. Box-and-whisker plots are presented as described in Fig. 11. Horizontal bars indicate statistically significant differences between groups (*p < 0.05, **p < 0.01)

**Fig. 13:**
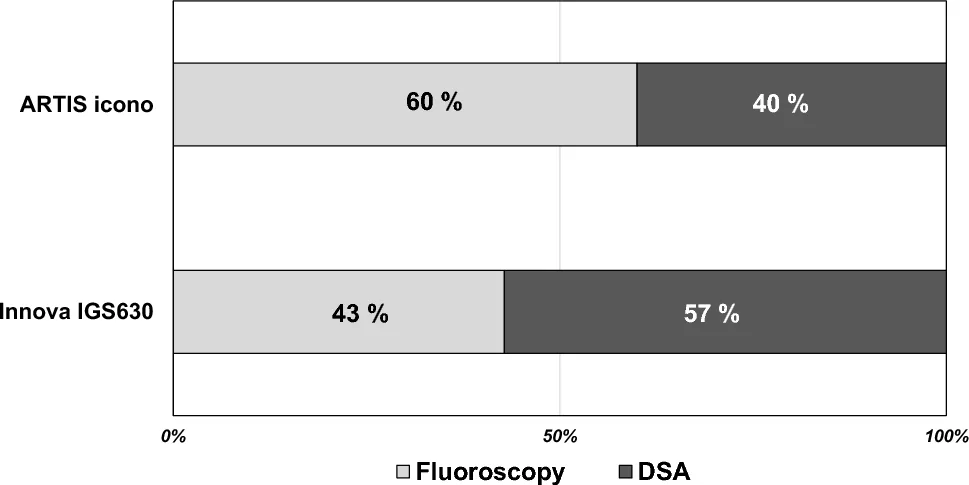
Relative contributions of fluoroscopy and DSA to the total patient entrance reference air kerma (Ka,r) for the new and conventional angiography systems

**Fig. 14:**
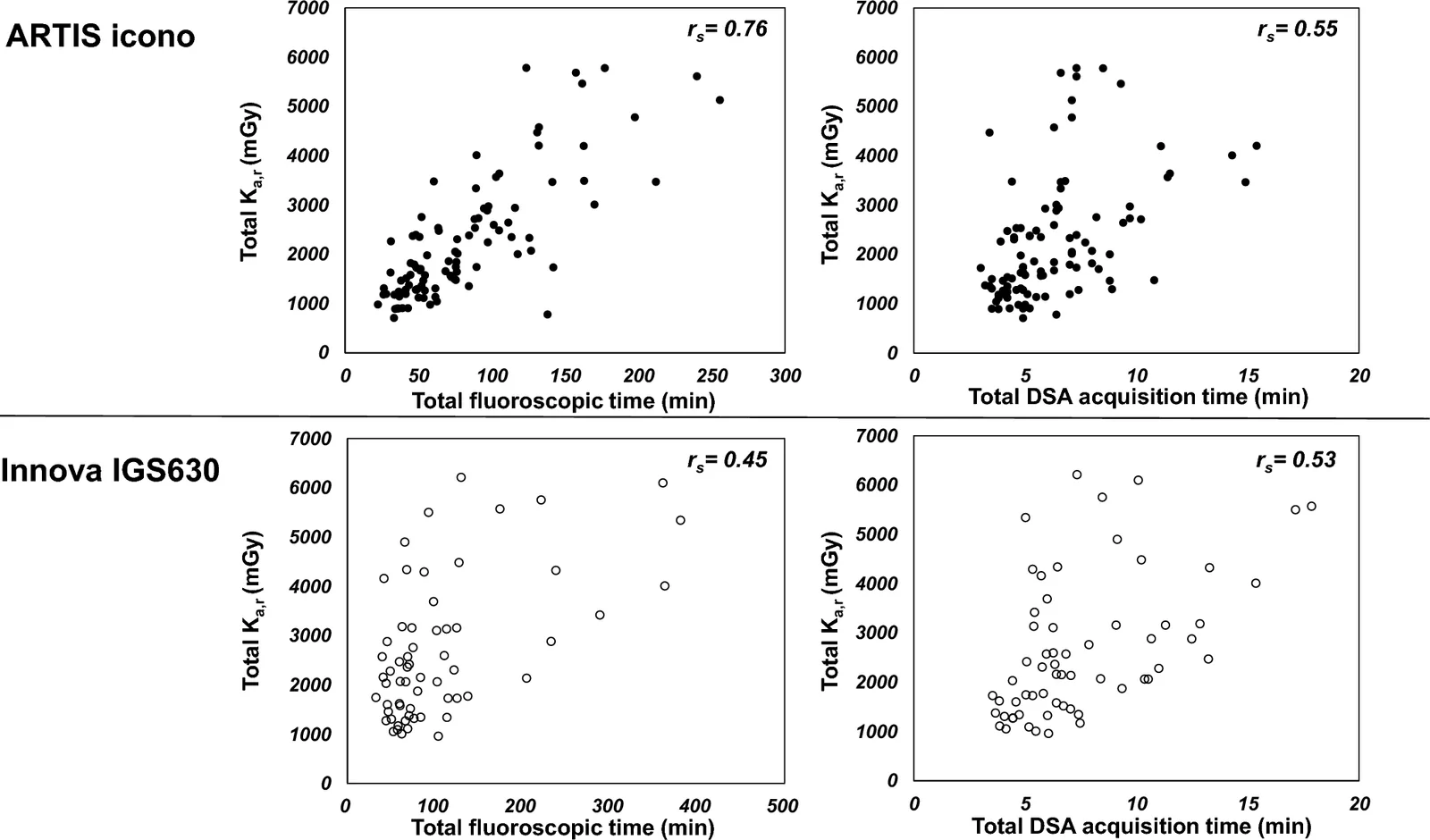
Correlations between total patient entrance reference air kerma (K_a,r_) and procedural parameters for the new and conventional angiography systems. The relationships between total K_a,r_ and total fluoroscopy time (left) and total DSA acquisition time (right) are shown. Spearman's correlation coefficients (r_s_) are indicated in each panel

Figure 12 presents the related parameters. The total fluoroscopy time was 71 [48–104] minutes for the new system and 74 [60–115] minutes for the conventional system (p = 0.07). The total DSA acquisition time was 5.7 [4.5–7.3] minutes for the new system and 6.4 [5.3–9.1] minutes for the conventional system (p < 0.05). The proportion of fluoroscopy to DSA within the total K_a,r_ is shown in Fig. 13. This ratio was 60–40% (fluoroscopy to DSA) for the new system, and 43–57% for the conventional system. The relationship between the total K_a,r_ and the related parameters is shown in Fig. 14. Total K_a,r_ showed significant positive correlations with both total fluoroscopy time and total DSA acquisition time in both systems (all p < 0.001). The correlation between total K_a,r_ and total fluoroscopy time (Spearman's correlation coefficient, r_s_; 95% CI) was r_s_ = 0.76 (95% CI, 0.65–0.84) for the new system and r_s_ = 0.55 (95% CI, 0.32–0.72) for the conventional system. The corresponding correlation with total DSA acquisition time was r_s_ = 0.55 (95% CI, 0.38–0.68) for the new system and r_s_ = 0.53 (95% CI, 0.28–0.70) for the conventional system.

## Discussion

### Reference dose

This study evaluated the reference doses for fluoroscopy and DSA at each FOV size for the new and conventional systems. The fluoroscopy dose rate at a diagonal FOV size of 12.6 inches for the new system was 14.5 mGy/min in the normal-dose mode and 10.5 mGy/min in the low-dose mode, which was 28% lower than that in the normal-dose mode. Additionally, the fluoroscopy dose rate in the low-dose mode of the new system was 10.5 mGy/min, compared with 10.3 mGy/min in the conventional system. The DSA dose at a diagonal FOV size of 12.6 inches for the new system was 1.04 mGy/frame in the normal-dose mode and 0.72 mGy/frame in the low-dose mode, which was 31% lower than that in the normal-dose mode. These findings suggest that noise reduction to maintain the target CNR may have contributed to the higher fluoroscopic dose because the new system determines the X-ray exposure parameters based on CNR. In contrast, they also suggest that the new system may maintain CNR at a lower radiation dose than the conventional system during DSA.

As with fluoroscopy, the roadmap dose rate decreased with increasing FOV size, whereas the estimated P_KA_ increased. Therefore, the choice of FOV involves a trade-off between reducing the maximum skin dose and increasing the cumulative radiation burden.

In this study, reference dose measurements were performed using a homogeneous 20-cm-thick PMMA phantom in accordance with the national guideline. However, because a homogeneous phantom does not provide contrast structures, it remains unclear how the CNR-based exposure control system adjusts X-ray exposure parameters under these measurement conditions. Accordingly, the present measurements should be interpreted as reference dose measurements performed under standardized dosimetric conditions rather than under clinically relevant image conditions. The European Federation of Organisations for Medical Physics (EFOMP) protocol for quality control of dynamic X-ray imaging systems (2024) recommends measuring dose rates using multiple PMMA thicknesses and explicitly indicates patient-equivalent PMMA thickness ranges for different clinical applications [49]. Therefore, in studies focusing on neuroendovascular procedures, additional dose measurements using thinner PMMA phantoms that more closely represent cranial anatomy may be warranted.

### Image quality evaluation

In this study, we evaluated three parameters—spatial resolution, low-contrast resolution, and dynamic range—of fluoroscopy and DSA images obtained from both new and conventional systems using a QC phantom. The image quality evaluation demonstrated that, for fluoroscopic images, the normal-dose mode of the new system showed higher image quality scores than the conventional system for all evaluation parameters (spatial resolution and low-contrast resolution, p < 0.01; dynamic range, p < 0.05). In addition, the low-dose mode showed higher scores than the conventional system for spatial resolution (p < 0.01) and low-contrast resolution (p < 0.05). For DSA images, both the normal-dose and low-dose modes of the new system showed higher scores than the conventional system for spatial resolution (p < 0.01) and low-contrast resolution (p < 0.05). These findings indicate that, under phantom conditions, the new system demonstrated improved image quality compared with the conventional system under a neurovascular imaging protocol, which is consistent with the phantom-based findings reported by Werncke et al. [38] using an abdominal imaging protocol. In particular, cerebral EVT requires higher spatial resolution and greater low-contrast detectability because fine intracranial vessels and low-radiopacity treatment devices must be visualized under fluoroscopic guidance [34, 39–41]. Therefore, the improved image quality demonstrated under phantom conditions may be beneficial for clinical neurovascular EVT, where high image quality is essential for device visualization and procedural guidance. However, because the phantom used in this study was static, the present findings may not fully reflect image quality during clinical neurovascular EVT, where image assessment is based on dynamic fluoroscopic and DSA images. Therefore, the results should be interpreted with caution.

Currently, the minimum diameter of guidewires used in clinical practice is 0.008 inch (0.2 mm). As shown in Table 4, this corresponds to the 0.2-mm wire in the 1.5-mm Cu (medium-contrast) region, which is assigned a spatial resolution score of 6 points. Therefore, a score of 6 may serve as a practical reference for the visual detectability of clinically relevant guidewires, although slight variations may occur depending on the material properties. Under the conventional system, the mean spatial resolution score for fluoroscopic images was 5.6 ± 0.5 points, indicating that guidewires of this diameter may not be visible under fluoroscopic conditions. In contrast, the mean spatial resolution scores obtained with the new system exceeded this reference in both the normal-dose mode (6.9 ± 0.7) and the low-dose mode (6.5 ± 0.5), suggesting that guidewires of this diameter would likely be visible under fluoroscopic conditions. There were no statistically significant differences in the image quality evaluation scores between the normal-dose and low-dose modes of the new system across all evaluation parameters for both fluoroscopy and DSA. This suggests that, while the use of the low-dose mode is somewhat less effective than the normal-dose mode, it allows for a reduction in the procedural radiation dose while enabling the visibility of the treatment devices. However, because this proposed reference was not validated by receiver operating characteristic (ROC) analysis, it should be considered exploratory.

As shown in Table 1, the new system is equipped with a detector featuring a smaller pixel size (154 μm vs. 200 μm) and a larger matrix size (2586 × 1904 vs. 1536 × 1536) than the conventional system. Jiang and Wilson reported that differences in flat-panel detector pixel size within the range of 150–200 μm have a direct and measurable impact on the visualization of stents and other fine interventional devices during interventional fluoroscopy [50]. The improved image quality scores observed in this study may therefore have been influenced not only by the exposure control system but also by differences in detector hardware.

As shown in Table 3, the displayed K_a,r_ during the phantom image quality evaluation was higher in the normal-dose mode of the new system than in the conventional system. Accordingly, the improved fluoroscopic image quality observed in the normal-dose mode may also reflect the higher radiation dose rather than improved dose efficiency alone. Notably, despite a lower displayed fluoroscopic dose during the phantom image quality evaluation than that of the conventional system, the low-dose mode still demonstrated higher scores for spatial resolution and low-contrast resolution, suggesting that the observed image quality improvement cannot be explained solely by increased radiation dose.

The relatively low ICC for dynamic range in DSA may reflect the relatively large residual variance compared with the between-subject variance. Systematic differences among raters appeared to be negligible, as indicated by the near-zero rater variance component. Furthermore, the small number of subjects (n = 3) may have limited the precision of the estimated between-subject variance.

In the present image quality evaluation, the KC-001 phantom was placed on a 10-cm-thick PMMA phantom. The displayed K_a,r_ differed by less than 10% from that obtained using a 20-cm-thick PMMA phantom under all fluoroscopic and DSA conditions. However, because the exposure control system automatically determines exposure parameters, including tube voltage and additional filtration, the X-ray beam quality may not have been identical between the two phantom configurations. Accordingly, the present evaluation reflects the overall image quality performance of each angiography system under clinically representative imaging conditions rather than under standardized beam quality conditions. Consequently, differences in X-ray beam quality may have influenced the observer-based image quality assessment.

### Clinical dose evaluation

The total K_a,r_ for EVT of cerebral aneurysms performed with the new system was 1818 [1301–2744] mGy, which was lower than the 2283 [1580–3184] mGy achieved with the conventional system (p < 0.05), representing a 20% reduction. Whereas the study by Werncke et al. [38] was limited to phantom experiments, the present study additionally evaluated radiation dose during clinical neurovascular EVT. Because the conventional and new systems represent different technological generations, the observed reduction in total K_a,r_ likely reflects the combined effects of multiple technical differences between the two angiography systems and cannot be attributed to the CNR-based exposure control mechanism alone. Furthermore, one possible explanation for the reduction in total K_a,r_ is that the improved fluoroscopic image quality demonstrated in the phantom-based image quality evaluation (Fig. 10) reduced the need for repeated DSA confirmation during device manipulation. This may have contributed to the shorter DSA acquisition time observed clinically (Fig. 12) and, consequently, to the reduction in total K_a,r._ However, because the image quality evaluation was based on a phantom experiment, this interpretation should be made with caution.

The total P_KA_ of the new system was 173 [120–237] Gy·cm^2^, exceeding that of the conventional system (137 [93–207] Gy·cm^2^; p<0.05), representing a 26% increase. K_a,r_ and P_KA_ represent different aspects of radiation exposure. K_a,r_ reflects the cumulative air kerma at the patient entrance reference point, whereas P_KA_ incorporates both air kerma and the irradiated field area. Therefore, a lower K_a,r_ does not necessarily result in a lower P_KA_ when a larger irradiated field is used. This physical difference may partly explain the apparently divergent findings of lower total K_a,r_ but higher total P_KA_ with the new system.

Chun et al. reported a mean dose–area product (DAP) of 226.0 ± 129.2 Gy·cm^2^ for the endovascular treatment of intracranial aneurysms [51]. In addition, Opitz et al. reported a median DAP of 157 Gy·cm^2^ (interquartile range, 108–217 Gy·cm^2^) [52]. In the present study, the median total P_KA_ with the new system was 173 Gy·cm^2^, which was within the range of these previously reported values. However, direct comparisons should be interpreted with caution because procedural complexity, treatment strategy, imaging protocols, and angiography system specifications differed among the studies.

One possible explanation for the higher total P_KA_ observed with the new system is the difference in magnification strategy during roadmap-guided device deployment. Magnified roadmap fluoroscopy is routinely required during device placement. Because the conventional system was not equipped with a digital zoom function, magnification was routinely achieved by reducing the FOV to 6.7 inches. In contrast, the new system typically used digital zoom while maintaining a 12.6-inch FOV. As described in Sect. 2.3, digital zoom in the new system was available only for roadmap images and was not applied during live fluoroscopy; therefore, collimation had to be adjusted manually. To maintain visualization of the guiding catheter during device manipulation, tight collimation outside the digitally magnified region may not always have been applied. This practice may not have been fully optimized during the transition to the new angiography platform, potentially resulting in irradiation of a larger area than necessary in some procedures.

Borota et al. demonstrated that optimized collimation using spot fluoroscopy can substantially reduce unnecessary radiation exposure during neurointerventional procedures [53]. Consistent with this interpretation, the estimated P_KA_ during roadmap fluoroscopy was 2.1 Gy·cm^2^/min with the conventional system using a 6.7-inch FOV, compared with 6.5 Gy·cm^2^/min with the new system using a 12.6-inch FOV (Fig. 8). Therefore, the higher total P_KA_ observed with the new system should not be interpreted as an intrinsic characteristic of the CNR-based exposure control system, but may instead reflect suboptimal magnification and collimation practices during the transition period. Further optimization should include appropriate FOV selection, tighter collimation during roadmap fluoroscopy, and continued operator education regarding field-size management. The higher total P_KA_ may reflect an increase in the cumulative radiation burden associated with stochastic effects and should therefore be interpreted carefully from the perspective of the ALARA principle [54].

In Japan, the DRL values for neurovascular EVT are defined as the total K_a,r_ and total P_KA_ for each disease category [55]. The most recent DRL values for cerebral aneurysm EVT, published in July 2025, are 2400 mGy for total K_a,r_ and 190 Gy·cm^2^ for total P_KA_. In the present study, the median total K_a,r_ and total P_KA_ for procedures performed using the new system were lower than these DRL values. However, because DRLs are intended as benchmarks for optimization rather than target or optimal dose levels, values below the DRLs do not necessarily indicate that radiation doses have been fully optimized. The International Commission on Radiological Protection (ICRP) has proposed the concept of the achievable dose, which corresponds to the 50th percentile of the dose distribution and serves as an additional benchmark for optimization [56]. In the 2025 Japanese DRLs, the 50th percentile values for cerebral aneurysm EVT are presented as 1900 mGy for total K_a,r_ and 150 Gy·cm^2^ for total P_KA_. In the present study, the median total K_a,r_ was close to the 50th percentile value, whereas the median total P_KA_ remained higher. Therefore, although the new system achieved radiation doses below the current DRLs, further optimization, particularly with respect to reducing P_KA_, may still be warranted.

Among the procedural parameters evaluated in this study, total fluoroscopy time showed the strongest correlation with total K_a,r_ in the new system (Spearman's correlation coefficient, r_s_ = 0.76; 95% CI, 0.65–0.84; p < 0.001), which is consistent with the findings reported by Hassan et al. [57]. Together with the increased contribution of fluoroscopy to the total K_a,r_ (Fig. 13), these findings suggest that optimization of fluoroscopy-related imaging parameters may represent an effective and efficient strategy for reducing K_a,r_ and, consequently, minimizing the risk of radiation-induced skin injury in patients. Furthermore, as shown in Fig. 10, no significant differences in image quality scores were observed between the normal-dose and low-dose modes of the new system in the phantom experiment. Although the clinical cases analyzed in this study were performed using the normal-dose mode, these findings suggest that clinical application of the low-dose mode may further reduce K_a,r_ while maintaining sufficient visualization of treatment devices.

In addition to patient radiation protection, optimization of procedural radiation dose is also important for reducing occupational radiation exposure in IR [58–60]. In particular, reducing lens radiation exposure among IR practitioners remains an important challenge [61–65]. Given the strong correlation between patient and operator radiation doses [66, 67], the clinical use of the low-dose mode may also contribute to reducing operator lens exposure. Future studies should include detailed assessments of occupational radiation exposure using real-time personal dosimeters.

## Limitations

This study has several limitations.

1. Retrospective, single-center design

First, this was a retrospective, single-center study, and the conventional and new angiography systems were evaluated during different time periods. Therefore, the findings may not be fully generalizable to other institutions, and temporal changes in operator experience, device technology, and procedural strategies may also have influenced the clinical results.

2) No multivariate adjustment

Second, multivariate analyses were not performed to adjust for potential confounding factors, such as patient characteristics, procedural complexity, and treatment techniques. Therefore, residual confounding may have influenced the clinical dose comparisons.

3) System comparison (hardware vs. CNR-based exposure control)

Third, this study compared complete angiography systems rather than the exposure control algorithms alone. Consequently, the respective contributions of the CNR-based exposure control mechanism and differences in detector hardware, including pixel size and matrix size, could not be distinguished.

4) Phantom-based image quality evaluation

Fourth, the image quality evaluation was based on a static QC phantom and homogeneous PMMA phantoms. Therefore, the results may not fully reflect dynamic clinical imaging conditions or the behavior of the CNR-based exposure control system under realistic anatomical conditions. Furthermore, objective CNR measurements were not performed; therefore, the present study evaluated overall image quality rather than CNR performance itself.

## Conclusion

This study evaluated the image quality and radiation dose following the transition from a conventional detector-dose-based to a new CNR-based angiography system using both phantom experiments and clinical neurovascular EVT data. Compared with the conventional system, the new angiography platform demonstrated superior phantom image quality and was associated with a 20% reduction in total K_a,r_ during cerebral aneurysm EVT. Although total P_KA_ increased by 26%, this increase may have reflected differences in magnification strategy and collimation practice, and the median total P_KA_ remained below the current Japanese DRL value. These findings suggest that the new system has the potential to reduce patient skin dose while maintaining high image quality. Further optimization of procedural techniques, particularly with respect to FOV selection and collimation practices, may enable additional reductions in cumulative radiation exposure.

## Data Availability

The datasets generated and/or analyzed during the current study are available from the corresponding author on reasonable request.
